# Professionals’ preferences in prenatal counseling at the limits of viability: a nationwide qualitative Dutch study

**DOI:** 10.1007/s00431-017-2952-6

**Published:** 2017-07-07

**Authors:** Rosa Geurtzen, Arno van Heijst, Jos Draaisma, Laura Ouwerkerk, Hubertina Scheepers, Mallory Woiski, Rosella Hermens, Marije Hogeveen

**Affiliations:** 1 Department of Pediatrics, Radboud University Medical Center Amalia Children’s Hospital, PO Box 9101, 6500HB Nijmegen, Internal Code 804, The Netherlands; 2 Department of Gynecology, Maastricht UMC+, Maastricht, The Netherlands; 3 Department of Gynecology, Radboud university medical center, Nijmegen, The Netherlands; 4 Scientific Institute for Quality of Care, Radboud university medical center, Nijmegen, The Netherlands

**Keywords:** Prenatal counseling, Limits of viability, Decision making, Extreme prematurity

## Abstract

Prenatal counseling practices at the limits of viability do vary, and constructing a counseling framework based on guidelines, professional and parental preferences, might achieve more homogeneity. We aimed to gain insight into professionals’ preferences on three domains of counseling, particularly *content*, *organization*, and *decision making* and their influencing factors. A qualitative, nationwide in-depth exploration among Dutch perinatal professionals by semi-structured interviews in focus groups was performed. Regarding *content* of prenatal counseling, preparing parents on the short-term situation (delivery room care) and revealing their perspectives on “quality of life” were considered important. Parents should be informed on the kind of decision, on the difficulty of individual outcome predictions, on survival and mortality figures, short- and long-term morbidity, and the burden of hospitalization. For *organization*, the making of and compliance with agreements between professionals may promote joint counseling by neonatologists and obstetricians. Supportive materials were considered useful but only when up-to-date, in addition to the discussion and with opportunity for personalization. Regarding *decision making*, it is not always clear to parents that a prenatal decision needs to be made and they can participate, influencing factors could be, e.g., unclear language, directive counseling, overload of information, and an immediate delivery. There is limited familiarity with shared decision making although it is the preferred model.

*Conclusion*: This study gained insight into preferred *content*, *organization*, and *decision making* of prenatal counseling at the limits of viability and their influencing factors from a professionals’ perspective.

**Table:** 

**What is Known:** *• Heterogeneity in prenatal counseling at the limits of viability exists* *• Differences between preferred counseling and actual practice also exists*
**What is New:** *• Insight into preferred content, organization, and decision making of prenatal periviability counseling and its influencing factors from a professionals’ perspective. Results should be taken into account when performing counseling.* *• Particularly the understanding of true shared decision making needs to be improved. Furthermore, implementation of shared decision making in daily practice needs more attention.*

## Introduction

Prenatal counseling at the limits of viability is an important but difficult task for perinatal professionals. To support them, several recommendations on counseling have been published in guidelines or as expert opinions [2, 7, 10, 15, 22–24, 32, 35, 41]. Opinions on how to perform prenatal counseling diverge among individual professionals [21, 22, 28]. Earlier, it has been demonstrated that actual prenatal counseling practices appear to be very heterogeneous, within and between countries [1, 6, 12–14, 34, 37, 50]. However, since the outcome of counseling has major impact on life or death decisions, practice variation is unwanted when it is not based on fetal or parental characteristics.

More homogeneity might be achieved by constructing a framework to support prenatal counseling at the limits of viability [24, 46]. Gaps between actual and preferred counseling by professionals appear to exist, as well as between professionals’ personal preferences and treatment guidelines, with regard to counseling and decision making [13]. For example, shared decision making (SDM) is suggested as preferred decision model in prenatal counseling by the AAP but not always performed [1, 16, 26]. To ensure support from professionals and applicability in daily practice of a framework, both qualitative and quantitative input on counseling preferences from stakeholders should be used. Research regarding prenatal counseling at the limits of viability using qualitative methodologies has been published and focused on parents [3, 19, 53], professionals [11, 51], or both [16, 26, 40, 44]. However, no in-depth exploration of known preferences in prenatal counseling among professionals was performed. For optimal counseling, this in-depth exploration of preferred counseling content, organization and decision making, and its influencing factors should be performed, from both professionals’ and parents’ perspective, and these should be included in a framework.

This study aims to gain insight into preferred *content*, *organization*, and *decision making* of prenatal counseling and their influencing factors from a professionals’ point of view.

## Materials and methods

### Study design and setting

We performed a qualitative study among Dutch perinatal professionals using semi-structured focus group interviews to explore in-depth the preferences in prenatal counseling. This study is part of the Dutch PreCo study (Prenatal Counseling in extreme Prematurity), which evaluates counseling at the limits of viability among perinatal professionals and parents in order to construct a framework. This study was initiated when the Dutch guideline for treatment at the limits of viability was changed in 2010 (clinicaltrials.gov NCT02782650 [42] & NCT02782637 [43]). All 10 level III centers for perinatal care in the Netherlands participated in the PreCo study.

### Study population

Focus group meetings (group interviews) were organized until saturation was achieved. By using various compositions (homogeneous and heterogeneous backgrounds, local and national groups), we tried to generate different types of discussions. For logistical reasons, we organized focus groups during existing national conferences or meetings. We aimed to have representatives of all 10 Dutch level III centers in at least one of the focus groups. Participants were approached by their colleagues, since we had a contact person (one obstetrician and one neonatologist) in every center for our PreCo study. Participants had to be (fellow) neonatologist or (fellow) obstetrician, we only excluded members of our study group.

### Data collection

The focus group interviews were conducted between May and July 2015. These interviews lasted between 50 and 80 min. Informed consent forms were signed and a short demographic questionnaire was filled out. The chairman (MH, project leader) started by explaining the process of the focus group interview. One or two observers attended each interview (RG, RH, HS). We performed semi-structured interviews using an interview guide based on prior results of the PreCo study*.* Printed forms showing results (tables and graphs) from the PreCo surveys were distributed and used as background information during the interviews. The interview guide contained three main domains of interest of counseling at the limits of viability: *content*, *organi*za*tion*, and *decision making.* Within these domains, several themes were included, for example when there was a mismatch between preferred and current counseling found in prior PreCo study results. For the first domain (content), the themes were specific preferred content, use of statistics, and potential ways of prioritizing topics. For the second domain (organization), the themes were joint counseling, use of supporting material, and use of protocols. For the third domain (decision making), the “acknowledgement that there is a prenatal decision to be made about active care versus comfort care” and SDM as preferred decision-model were the themes included. Interview questions were open ended and designed to further explore these themes and to find potential influencing factors.

### Analysis

All focus group interviews were audio-taped and literally transcribed (RG or LO). Next, two researchers independently analyzed all transcripts, and quotes were classified according to the corresponding themes within the three domains (RG and LO). Thereafter, these quotes were coded into summarizing terms. For example, in the domain *organization*, one theme was the “use of supportive material” wherein several quotes were found such as “we will counsel more uniform when using a decision aid,” then the term “uniformity” was made. All discrepancies were discussed until consensus was reached (RG, LO, MH, RH). The analyses were conducted with the aid of the qualitative analysis tool ATLAS.ti GmbH Version 7.1.5 (Berlin, Germany).

## Results

### Demographics

Four focus groups meetings (consisting of 5 to 12 participants per group) were organized. One focus group contained both obstetricians and neonatologists; the other groups included either obstetricians or neonatologists. Three focus groups were national (a mix from several centers); the fourth was local (one center only). A total of 35 participants (23 neonatologists, 12 obstetricians) were included, all level III centers were represented by at least one person. Years of experience ranged from 2 to 40 years, age ranged from 36 to 63 years.

### Domain: content of prenatal counseling

**Fig. 1:**
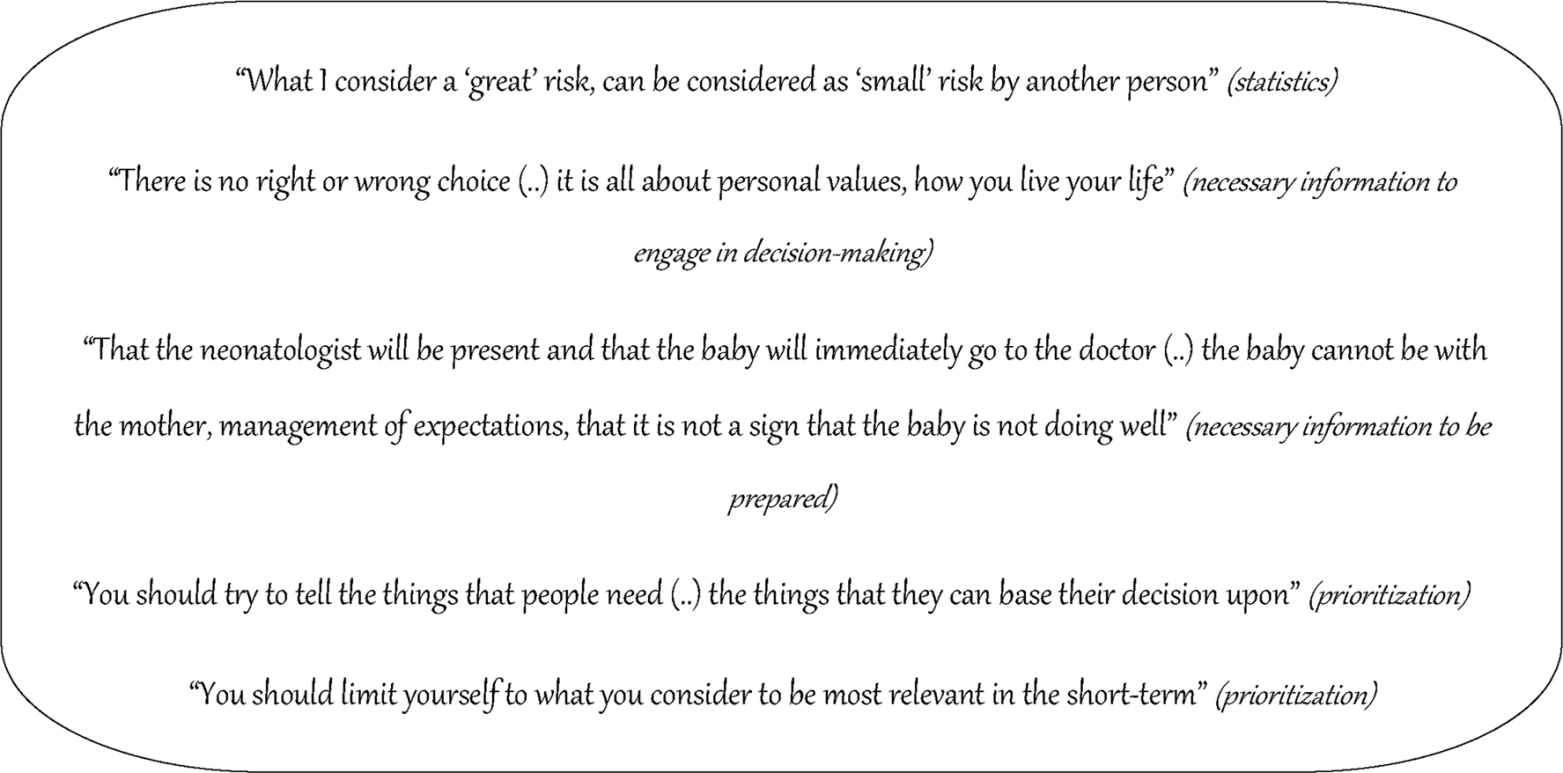
Domain: content of prenatal counseling—quotes on several themes associated with preferred content

Table 1 shows the different themes in the domain of content with their corresponding terms and illustrative quotes can be found in Fig. 1. Regarding the use of statistics, participants mentioned that uniform figures can assure more similarity between professionals. However, concerns were expressed on the validity of the statistics: They are variable over time and cohort dependent and do not predict an individual outcome. For the individual parent, participants mentioned that statistics may help to provide insight, and so value judgments on outcome data can be left to the parents (e.g. one third chance can be regarded as acceptable by one, and as substantial by the other). Next to these stated (dis)advantages, the specific preferences regarding the use of statistics can be found in Table 1.

**Table 1:** Domain: content of prenatal counseling—terms associated with preferred content of counseling

Theme	Terms
Statistics/outcome data	Use general outcome or ranges, without excessive detail Translate numbers to an understandable level Use most recent Dutch outcome date for short term, international for long term Leave value judgment of odds to parent(s) Explain general outcome statistics versus individual prognosis Explain the denominator (e.g. what is a handicap)
Necessary information for parents to engage in decision making	No right or wrong choice Uncertainty of predictions Parents’ perspective - quality of life, - valuation of disabilities Short term morbidity Intact survival versus long term morbidity - odds for disabilities, - severity, impact on parents, - labeling handicaps Survival and mortality = Suffering of the newborn during admission, proportionality Multiple decision moments will follow - for parents and for doctors, - switch of legal responsibility for medical decision making from parent (prenatal) to doctor (postnatal) - sometimes there will be nothing to choose Emphasize the decision moment before birth Parents’ expectations - adjust outcome predictions, - no guarantees (not able to predict course independent of decision) - an infant can be born alive despite a comfort care decision Check for understanding
Necessary information for parents to be prepared for the near future	Practical information on direct delivery room care - delivery mode (C-section), - who is present at delivery - support of transition takes time in delivery room before mother can see the baby, - baby will not stay with mother and must go to NICU, - immediate breathing issues, - first impression on baby’s state, - appearance (in plastic bag, with IV) - father’s role First NICU hours Tour at NICU when possible Parents’ expectations - multiple decision moments, - maintainability of choice for active care, - active care is not the same as to continue at all costs - sometimes no “return” despite worse prognosis, - baby can live for some time when deciding for comfort care, - goal of treatment = quality of life Predictability Transfer when lack of space Mortality: the baby can die Long term morbidity - mental retardation, - cerebral palsy Intubation, ventilation IVH/cerebral bleeding Infection Impact on family, relationship Social work Bonding with child (parents’ contribution)
Prioritization of topics in counseling	Key topics based on goal of parental engagement in decision making - mortality - long-term morbidity Key topics based on parental characteristics - parents’ expectations - quantity of information parents will and can handle (IQ, EQ) - (mis)interpretation/assumptions on decisions - parents’ norms and values - language (understanding) - religion and culture - existing knowledge on prematurity, disabilities Key elements based on goal of preparing the parents - short term issues (first days of life) Depending on circumstances - medical setting - time pressure - presence of partner Depending on whether a decision has already been made

Exploration of essential information for parents to support decision making revealed several general advices. Parents should be told that there is an important decision to make, that there is no right or wrong decision, and that it is hard to make outcome predictions for their individual baby. Furthermore, revealing expectations of the parents and their perspective on quality of life were mentioned as important. An explanation of (intact) survival and mortality figures, short- and long-term morbidity, and the burden of a NICU period should be provided. When participants were asked what essential aspects should be explained to parents to optimally inform them about the nearest future, they mentioned delivery room management and medical risks during the first days of life. Also, many participants suggested a NICU tour before delivery, when possible.

Various ways of prioritizing all these potential topics in counseling were mentioned. Participants preferred to prioritize based on the decision (initiating care or not) that has to be made and/or based on parental characteristics, and/or on the preparation of the parents on what to expect in the nearest future.

### Domain: organization of prenatal counseling

**Fig. 2:**
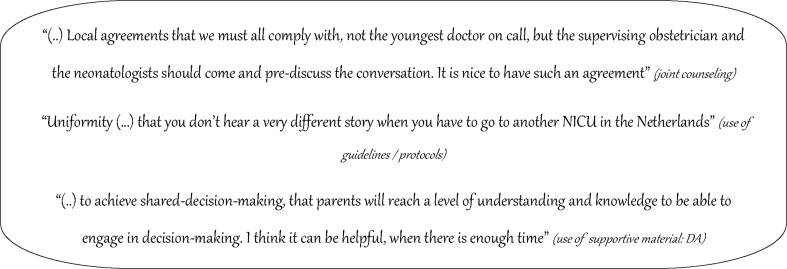
Domain: organization of prenatal counseling—quotes on several themes associated with preferred organization of counseling

Table 2 shows the themes associated with preferred counseling in the “organization” domain and illustrative quotes can be found in Fig. 2. Participants mentioned that joint prenatal counseling by both the obstetrician and neonatologist can be facilitated when both groups make local agreements and comply with them, solve logistical issues, and share the responsibility for the counseling. There should, however, be sufficient staff, also during service hours. Patient-related logistic factors can influence the time available to counsel.

**Table 2:** Domain: organization of prenatal counseling—terms associated with preferred organization of counseling

Theme	Terms
Preference in the prenatal counseling at the limits of viability	Influencing factors
Joint prenatal counseling by both obstetrician and neonatologist	Patient related - partners’ presence - right amount of interlocutors - amount of time to delivery Prioritization and responsibility professionals Logistical issues - matching schedules between specialties - workload - planning Capacity staff (service hours) Decision made or not before dialog between specialties Extra: having a nurse joining the counseling conversation
The use of guidelines/frameworks/protocols	Personalization based on - medical characteristics - parental characteristics - preferred input of parents in decision making - preferred amount of information - preferred use of statistics/outcome data Box-checking character Feasibility Legal implications Uniformity (within and between centers) Neutrality Effectiveness Teaching applications Adherence to instructions/guideline as a rule
The use of supportive material in general	Availability material Quality material (im)personalization Availability up-to-date, applicable outcome statistics Reread information Uniformity/intercenter + interpersonal variability Neutrality Additive to conversation
The use of supportive material: decision aid	Visualization of complex information Increasing knowledge to joint decision making Time investment Reliable source of information Uniformity Neutrality Memorize and reread False feeling of one right decision Potential wrong decision General outcome statistics vs. individual prognosis

The preference of having a framework for prenatal counseling was influenced by several factors. Uniformity and neutrality (being non-directive) were mentioned as essential benefits. Most concerns seemed to exist about the possibility of personalization; a framework should allow personalization towards the specific situation (e.g. based on medical and parental characteristics, parental preferred input in decision making).

Regarding the use of supportive material (such as a leaflet, or a decision aid (DA)), the availability of suitable material was stated to be an important influencing factor. For reasons of uniformity, neutrality, and re-reading, participants would appreciate such material. Specific benefits for the use of a decision aid were the visualization of the decision and ensured parental involvement in decision making. Participants suggested up-to-date statistics, personalized baseline information, visualized information, and specified disabilities to be included in a DA. Finally, it should not be a checkbox replacing the conversation. Conditional on these recommendations, professionals were positive about using supportive material in general and specifically a DA for counseling and decision making at the limits of viability.

### Domain: decision making in prenatal counseling

**Fig. 3:**
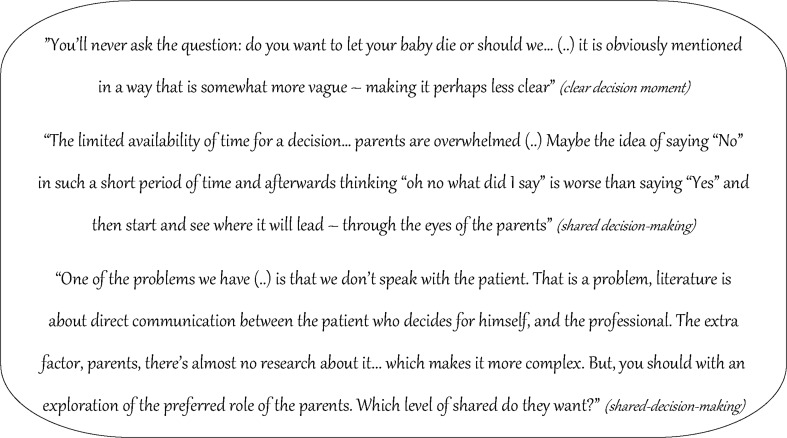
Domain: decision making of prenatal counseling—quotes on several themes associated with preferred decision making

Table 3 shows the themes associated with preferred counseling in the decision making domain and illustrative quotes can be found in Fig. 3. Suggestions were made to assure that it is always clear to parents that a prenatal decision should be made at 24 weeks GA. These included to mention this decision very explicitly, to specifically ask parents for their preference, and to check whether parents want to be involved in decision making.

**Table 3:** Domain: decision making in prenatal counseling—themes associated with preferred decision making

Theme	Terms
Preference in the prenatal counseling at the limits of viability	Influencing factors
It must be clear to parents that there is a decision moment	Doctor-related - (non)-directive counseling ((not) mentioning the decision) - (un)clear language - decision already made by obstetrician before neonatologist is involved Parent-related - whether parents want to be engaged in decision making - recall bias - potential overload information - whether parents already made a definite decision before conversation Organization-related - availability of time (immediate delivery) - availability of a counseling conversation
Shared decision making as preferred decision model	- several assumptions and definitions about SDM - co-existing support for other decision models - information-bias before counseling conversation - whether decision is already made before counseling conversation - surrogate decision-makers (parents) - (lack of) enough evidence based information - (lack of) enough time for SDM - resistance to SDM due to personal preference of the doctor for either comfort care or active care - emotions or subjectivity of the doctor

SDM was mentioned as the preferred counseling model at the limits of viability; however, it was not clear to what extent the concept of SDM was understood. Focus group members were asked for their definitions of SDM, which revealed a variety of definitions; see box 1.

**Box 1:** Different definitions of professionals on SDM

“well-informed parents saying what they want for their child, a decision which you can support as a professional. That both support the decision” “informed consent, because parents make their decision based on your counseling” “To both (parents and doctor) come to the same decision, matching the values of the patient and matching the professional standards” “Parents are deciding completely, you do not need to agree as a doctor” “50% vote for doctor and parent” “directive counseling” “SDM is no directivity” “I do not know what SDM is” “To inform as good as possible, understandable language on parental level, with a joint decision” “To be equivalent in the decision making. However, that will not be the case, you should inform parents and allow them to decide in freedom whatever matches with them”	

Next to giving definitions of SDM, professionals thought that many of them might not understand the meaning of other decision models. Doctors might switch between decision models (SDM, informed and paternalistic model), either within one case at different moments or between different cases based on that specific situation. Other influencing factors are found in Table 3. When exploring the decision making process and the preferred roles of parents and professionals therein, several goals were defined, such as to reveal expectations. More goals are found in Table 4.

**Table 4:** Preferred roles of parents and doctors in decision making, according to perinatal professionals

Preferred role of parents and doctor in decision making, according to perinatal professionals	
Preferred role parent in decision making	To make clear whether they want to be involved in decision making
	To make clear how disabilities are valued
Preferred role doctor in decision making	To reveal expectations
	To check understanding of information
	To make sure that decisions can be revised
	To provide neutral insight into survival with or without disabilities
	To make explicit whether parents want a role in decision making
	To inform that no precise outcome predictions are possible (general statistics vs. individual prognoses)
	To protect parents against unrealistic expectations
	To reveal what parents values are in life and what parents need (from the doctor) to engage in decision making (doctors role is not to have 50% input!)
	To explore and check a decision that was already made
	To explicitly inform that a prenatal decision needs to be made

## Discussion

This is the first nationwide study aiming to gain insight into preferred *content*, *organization*, and *decision making* of prenatal counseling at the limits of viability and its influencing factors from a professionals’ perspective through qualitative research. With this information, a framework to support prenatal counseling at the limits of viability can be developed, to achieve more homogeneity in this difficult area.

Regarding *content* of prenatal counseling, preparing parents on the short-term situation (delivery room care) and revealing their perspectives on “quality of life” was considered important. Parents should be informed on the kind of decision, on individual predictions being difficult, on survival and mortality figures, short- and long-term morbidity, and the burden of hospitalization. Various ways of prioritizing this multitude of topics exist. For *organization*, joint counseling by neonatologist and obstetrician was often preferred. The making of and compliance with agreements between professionals can promote this. Supportive materials were considered useful but only when up-to-date, in addition to conversation and with possibility for personalization. Regarding *decision making*, it is not always clear to parents that a prenatal decision needs to be made and that they can participate. Influencing factors could be, e.g., unclear language, directive counseling, overload of information, and an immediate delivery. There is limited familiarity with shared decision making even though it is the preferred decision model.

### Domain: content of prenatal counseling

Professionals indicated that many topics are important to discuss in prenatal counseling at the limits of viability, consistent with literature [1, 7, 15]. Since time can be limited and parents simply will not remember everything [27], priorities must be set. These appeared to vary between professionals and will influence the selection of topics. The majority agreed that making the decision on initiating care was the most important goal of prenatal counseling, but other ways of prioritizing were also mentioned (preparing the parents for the near future, or selecting topics based on parental characteristics). In 2005, Bastek showed that a majority of neonatologists (58%) saw their primary role during the prenatal consultation as providing factual information to the parents. Far fewer (27%) thought that their main role was to assist the parents in weighing the risks and benefits of various management options. Grobman and Keenan suggested that the focus experienced by parents in their counseling conversations has not always been the decision making [16, 26]. As Watson appoints, within the “gray zone of viability,” the focus of prenatal counseling should be the decision making, and beyond the gray zone, the focus should shift to helping parents prepare [52]. The American Association of Pediatrics (AAP) also states that decision making is the primary focus of prenatal counseling at the limits of viability—to which we agree [7]. Thereby, this decision making involves more than medical factors, it is of utmost importance to be empathic, provide support, and give parents hope during the counseling [3, 30, 40, 49].

Regarding the topics of counseling, participants mentioned necessary information for the parents to be prepared for the near future (in Table 1). Remarkably, the resulting terms assume an active care decision. However, it is of utmost importance to also prepare parents on what can happen after a choice for comfort care. Moreover, focusing on consequences of active care only may put unwanted emphasis on that option, and neutrality towards the prospective parents can be lost.

### Domain: organization of prenatal counseling

Among other logistical issues, poor sense of responsibility, understaffing, and patient-related factors were mentioned as barriers to joint counseling. Local agreements between both professions involved, who share the responsibility for joint counseling and who both can give priority to this, were suggested to facilitate joint counseling. The department should be equipped for this: matching schedules and no understaffing, including during service hours. The Dutch guideline does recommend transfer to a tertiary center at 23^+4/7^ weeks GA to allow sufficient time for (repeated) counseling within 24 h in the tertiary center [8]. Given the barriers mentioned, this guideline apparently provides insufficient support for daily practice.

The use of protocols or frameworks in prenatal counseling has been suggested regularly [2, 7, 15, 23, 24] but is also viewed with skepticism [21, 28]. We revealed several influencing factors on a potential framework for counseling such as feasibility, uniformity within and between centers, and the potential for personalization, comparable to the benefits and disadvantages from literature [15, 21, 22, 24, 28]. The benefit of a counseling framework for teaching had also been recognized before [36]. Personalization in counseling is important and should be based on medical factors, parental factors, preferred input of the parents in decision making, the amount of preferred information, and the latest outcome data. When these criteria could be met, a counseling framework was considered to achieve more uniformity (less variation) and neutrality (less paternalism). The AAP as well suggests that written policies and procedures can promote consistent, timely, and effective counseling [7], and they also promote personalization in delivery room management based on fetal and maternal conditions and risks, as well as on parental beliefs regarding the best interest of their child.

An explanation for the discrepancy in preferred versus current use of supportive material appeared to be the lack of available, suitable material. Supportive material can be useful in prenatal counseling, either as written information [38] or as a DA [17, 18, 25]. The potential impossibility to personalize and to use up-to-date statistics raised concerns to our participants. Grobman found similar concerns since only 15% of the physicians asked for written material because they were concerned that clinical conditions could change so rapidly that static resources would not be effective [16]. However, that should not be a reason for not using material. Material can be personalized by doctors, for example by simply underlining and outlining what is of more or less relevance regarding the (medical) situation of the infant and wishes of the parents. Furthermore, cross-cultural differences in treatment-guidelines, language, and outcome data should encourage local institutions to develop their own material based on the positive experiences described [17, 18, 25, 38, 39].

### Domain: decision making in prenatal counseling

SDM is the preferred decision-model in prenatal counseling. We identified several barriers on SDM, such as the limited knowledge on what SDM actually is, limited availability of time and surrogate decision making. Some of these barriers are, to our opinion, misconceptions regarding SDM and may be improved by increasing knowledge and understanding. Others are harder to influence (such as an immediate delivery, surrogate decision making). However, we must aim at optimizing the circumstances to perform SDM as best as possible.

#### Limited knowledge on SDM

Limited knowledge on SDM had already been encountered by Makoul in 2006 [33] showing the use of various SDM definitions in literature. After conducting our focus group interviews, Stiggelbout published a key paper in which four steps of SDM were explained in a practical manner based on known literature such as Makoul and Elwyn [9, 33, 48]. The first step (1) is the professional informing the patient that a decision is to be made and that the patient’s opinion is important, in the second step, (2) the professional explains the options and their pros and cons, in the third step, (3) the professional and the patient discuss the patient’s preferences and the professional supports the patient in deliberation, and in the final fourth step, (4) the professional and patient discuss the patient’s wish to make the decision, they make or defer the decision, and discuss follow-up. In prenatal counseling, parents act as surrogate decision makers for their unborn child. According to our results, the understanding of SDM needs to be improved, although the preferred roles of parents and doctors in decision making included some aspects of SDM. Implementation of these concepts into daily practice may take time. The use of Stiggelbouts’ definition will be helpful. The fact that a prenatal decision needs to be made is not always recognized (step 1); this is influenced by several doctor-, patient-, and organization-related factors. Whether a decision has already been made before the counseling conversation (either by another doctor or by parents themselves) is one factor. We believe that it is still necessary to check how the decision was made. Steps 3 and 4 are important—simply asking whether parents want to be involved in decision making is not enough. The fourth step allows for various preferences in the extent of involvement that parents prefer, but it will still be a shared decision and parents will be involved. Even when parents want the professional to decide, the professional should take parental preferences/values into account—obtained by adequately performing step 3. But, professionals do have to check the preferred involvement of parents in the decision making, since they are known to be not good enough predicting this [54]. Furthermore, it is known that the perception of a shared decision is associated (in the long term) with lower grief scores compared to informed or paternalistic decision making [5]. The knowledge on SDM should be improved, and educational sessions might be helpful and will be performed. Furthermore, decision aids have been proven useful in SDM and will help both parents and professionals performing SDM [17, 18, 25, 47]; in the future, we will develop a Dutch decision aid on this topic as well.

#### Limited time for SDM

The time-issue is twofold. First, limited availability of time to counsel (due to an immediate delivery) is an issue as recognized before [7, 20] which cannot always be influenced. However, logistic circumstances must be optimized (timely referral to a tertiary center, 24/7 availability of perinatal professionals). Second, performing SDM itself was assumed to be (too) time-consuming. This is not proven; Legare stated that SDM does not take substantial more time that other counseling policies [29]. Moreover, even if SDM is more time-consuming, we think that this is justified considering the tremendous short- and long-term consequences of a birth at the limits of viability (coping with a NICU stay, complications, grief, etc.).

#### Surrogate decision making

Classic SDM is described for patients who decide for themselves. A model for pediatrics is non-existent [45]. In prenatal counseling, parents are seen as natural surrogates for their children. Prenatal decision making by a surrogate is even different from adult surrogate decisions, for example because information on patients past decisions and behavior is nonexistent and cannot serve as a reference to guide decisions [4, 15, 31].

### Strengths and limitations

This study is nationwide; all Dutch level III centers were included, and it is, to the best of our knowledge, the first qualitative study specifically exploring preferences in prenatal counseling and its influencing factors, needed for construction of a supportive framework. The use of interviews had the advantage of exploring complex phenomena and discovering new influencing factors. However, since this is a qualitative methodology, we do not have information to explicitly quantify the results. Furthermore, interpretation of interviews can be subject to bias. Therefore, we analyzed all transcriptions with two researchers independently. Another limitation is the national setting—making it uncertain to what extent the results apply internationally. However, many of the factors identified are not specifically related to the Dutch setting, and guidelines have similar aspects worldwide, so the results of this study can be relevant for international colleagues. Therefore, despite these limitations, we believe our work provides necessary insight into counseling at the limits of viability.

### Conclusions and future perspectives

This study gained insight into preferred *content*, *organization*, and *decision making* of prenatal counseling at the limits of viability and its influencing factors from a professionals’ perspective through qualitative research. The next step will be to reveal the preferences from parents. Combining the points of view from both professionals and parents, a framework to support prenatal counseling at the limits of viability will be developed, to achieve more homogeneity in this difficult area. Improving the knowledge on the shared decision making concept by perinatal professionals will be necessary.

## Abbreviations

AAP: American Association of Pediatrics
DA: Decision aid
GA: Gestational age
NICU: Neonatal intensive care unit
SDM: Shared decision making
